# Mouse cell line authentication

**DOI:** 10.1007/s10616-013-9545-7

**Published:** 2013-02-22

**Authors:** Jamie L. Almeida, Carolyn R. Hill, Kenneth D. Cole

**Affiliations:** 1 Biosystems and Biomaterials Science Division, National Institute of Standards and Technology, 100 Bureau Drive MS8312, Gaithersburg, MD 20899 USA; 2 Biomolecular Measurement Division, Applied Genetics Group, National Institute of Standards and Technology, 100 Bureau Drive MS8316, Gaithersburg, MD 20899 USA

**Keywords:** Mouse cell line, Authentication, Short tandem repeat, Multiplex PCR, Capillary electrophoresis, Genotyping

## Abstract

The scientific community has responded to the misidentification of human cell lines with validated methods to authenticate these cells; however, few assays are available for nonhuman cell line identification. We have developed a multiplex polymerase chain reaction assay that targets nine tetranucleotide short tandem repeat (STR) markers in the mouse genome. Unique profiles were obtained from seventy-two mouse samples that were used to determine the allele distribution for each STR marker. Correlations between allele fragment length and repeat number were determined with DNA Sanger sequencing. Genotypes for L929 and NIH3T3 cell lines were shown to be stable with increasing passage numbers as there were no significant differences in fragment length with samples of low passage when compared to high passage samples. In order to detect cell line contaminants, primers for two human STR markers were incorporated into the multiplex assay to facilitate detection of human and African green monkey DNA. This multiplex assay is the first of its kind to provide a unique STR profile for each individual mouse sample and can be used to authenticate mouse cell lines.

## Introduction

Cell line authentication is now required by certain journals prior to publication (Reid 2011; Barallon et al. 2010) and in some cases be mandatory before receiving funding from small granting agencies (Perkel 2011). The FDA has also instituted a requirement for the authentication of cell lines used to produce pharmaceuticals in their General Requirements for Laboratory Controls and the General Standards for Biological Products (21 CFR 211.160 (b) and 21 CFR 610.18 (b)). There are methods in place for authenticating human cell lines using multiplex PCR assays that target short tandem repeat (STR) markers in the human genome and are capable of generating a unique individual genotypic profile (Stacey et al. 1992; Masters 2001). Cell repositories are now genotyping their human cell lines using at least eight human STR markers including a marker for amelogenin, the sex identification locus (Castro et al. 2013; Perkel 2011). Large databases of STR profiles are available to confirm genotypes of human cell lines (Dirks and Drexler 2011) and provide a record of previously misidentified or cross-contaminated human cell lines (Capes-Davis et al. 2010). An updated list of misidentified human cell lines compiled by Capes-Davis and Freshney can be found on the American Type Culture Collection (ATCC) website (last updated on 9/3/2012 for version 6.8): http://standards.atcc.org/kwspub/home/the_international_cell_line_authentication_committee-iclac_/Database_of_Cross_Contaminated_or_Misidentified_Cell_Lines.pdf. The ATCC Standards Development Organization has recently published a consensus standard “Authentication of Human Cell Lines: Standardization of STR Profiling” (ANSI/ATCC ASN-0002-2011). Although there are successful methods in place for human cell line authentication, methods for nonhuman cell lines are not well established.

Mouse cell lines are the most common model system used to study human genes and disease (http://ec.europa.eu/research/health/pdf/summary-report-25082010_en.pdf). Mouse cells are used in the biomanufacturing of recombinant proteins (Barnes et al. 2000) and also function as feeder cells for embryonic stem cells (Eiselleova et al. 2008). Current techniques to identify mouse cell lines or mouse strains include microsatellite markers (simple sequence length polymorphism (SSLP) or STR markers) (Witmer et al. 2003; Matin et al. 1998; Schalkwyk et al. 1999; Zuo et al. 2012), single nucleotide polymorphisms (SNPs) (Tsang et al. 2005; Yang et al. 2009), and species specific primers (Higgins et al. 2010; Steube et al. 2008). *Mus musculus domesticus* mice, one of the most commonly used laboratory strains, are difficult to genotype due to many shared alleles (Schalkwyk et al. 1999; Witmer et al. 2003) resulting from extensive breeding. Reports have been published of multiplexing mouse SSLPs post-PCR by pooling the amplified products to distinguish between different strains of inbred mice (Witmer et al. 2003); however, most of the microsatellite markers that have been used for these purposes are dinucleotide in nature (Dietrich et al. 1996), mainly CA repeats, which result in noisy stutter and have higher mutation frequencies when compared to tetranucleotide repeats (Lee et al. 1999). The reduced stutter associated with tetranucleotide repeats allows for easier interpretation of single and mixed profiles (Butler 2001). Current methods lack the resolution to differentiate between individual mice of the same subspecies. SNPs are well conserved between inbred mice of the same strain making it difficult to differentiate between interstrain mice using this method. Even an extensive array containing over 600,000 mouse SNPs (Yang et al. 2009) is still unable to identify individual mice within the same subspecies. Species specific primers have been used to determine the origin of species for cell lines (Higgins et al. 2010; Steube et al. 2008; Holder and Cooper 2011); however, they lack specificity to identify down the individual level. This report describes an assay that can be used to authenticate mouse cell lines resulting in unique profiles for individual mouse samples based on tetranucleotide repeats that are stable with high passage number in the two different cell lines tested.

## Materials and methods

### Selection of short tandem repeat markers

Target STR markers were chosen for each chromosome, including the X and Y, by searching for tetranucleotide repeat sequences (AGAT and TCTA) of varying number of repeating units within the National Center for Biotechnology Information (NCBI) mouse genome build 38.1 using the BLAST program (http://blast.ncbi.nlm.nih.gov/Blast.cgi). Chromosome locations and GenBank accession numbers for the STR markers are listed in Table 1. Primers were tested to meet the following requirements: the locus must be present in every sample tested, the locus must contain a tetranucleotide repeat, and primers for each marker must amplify products in a functional multiplex. Two markers were located on mouse chromosome six; however, they are 90 Mb apart and on opposite arms of the chromosome and will be considered unlinked. In humans, markers that are over 50 Mb apart can be considered unlinked (Butler 2005b). Two well characterized human STR markers, D8S1106 and D4S2408, were included in the multiplex assay to screen for contamination of mouse cell lines. Both human STR markers have been previously used to identify human and African green monkey cell lines (e.g. Vero cells) (Hill et al. 2009; Almeida et al. 2011).

**Table 1:** Primers for STR amplification and corresponding chromosomal locations

STR marker	GenBank accession #	Location on chromosome (bp)	Primers (5′–3′)	Primer μM
18-3	NT_039674.8	60271556–60271705	F: [FAM]-TCTTTCTCCTTTTGTGTCATGC R: GTTTCTTGCTAAATAACTAAGCAAGTGAACAGA	0.200
4-2	NT_187032.1	82068280–82068580	F: [FAM]-AAGCTTCTCTGGCCATTTGA R: GTTCATAAACTTCAAGCAATGACA	0.125
6-7	NT_039353.8	51601265–51601685	F: [FAM]-AGTCCACCCAGTGCATTCTC R: GTTTCTTCATGTGGCTGGTATGCTGTT	0.075
9-2	NT_039474.8	74395400–74395000	F: [VIC]-GGATTGCCAAGAATTTGAGG R: GTTTCTTTCCTGAGTTGTGGACAGGGTTA	0.080
15-3	NT_039617.8	4930200–4930500	F: [NED]-TCTGGGCGTGTCTGTCATAA R: GTTTCTTTTCTCAGGGAGGAGTGTGCT	0.060
6-4	NT_039360.8	142021975–142022270	F: [NED]-TTTGCAACAGCTCAGTTTCC R: GTTTCTTAATCGCTGGCAGATCTTAGG	0.100
12-1	NT_039548.8	38480950–38481170	F: [VIC]-CAAAATTGTCATTGAACACATGTAA R: GTTTCTTTCAATGGTCAAGAAATACTGAAGTACAA	0.200
5-5	NT_109320.5	112641540–112641820	F: [PET]-CGTTTTACCTGGCTGACACA R: GTTTCTTGGTTTAAAACTCAATACCAAACAA	0.300
X-1	NT_039706.8	110959842–110960080	F: [PET]-GGATGGATGGATGGATGAAA R: GTTTCTTAAGGTATATATCAAGATGGCATTATCA	0.300
**D8S1106**	**NT_167187.1**	**12835860–12836150**	F: [VIC]-GTTTACCCCTGCATCACTGG R: GTTTCTTTCAGAATTGCTCATAGTGCAAGA	0.150
**D4S2408**	**NT_006316.16**	**31304210–31304514**	F: [NED]-TCATTTCCATAGGGTAAGTGAAAA R: GTTTCTTGCCATGGGGATAAAATCAGA	0.200

Mouse chromosomal locations (bp) are based on the current NCBI 38.1 mouse build. The chromosomal locations for human STR markers D8S1106 and D4S2408 (in bold) are based on the current NCBI 37.3 build. Primer concentrations listed are final concentrations of forward and reverse primers in a 20 μL reaction volume. Primer concentrations were determined empirically based on peak height, DNA concentration, and number of cycles in the PCR program

### Primer design

Primer3 software (http://fokker.wi.mit.edu/primer3/input.htm) was used to design PCR primers to flank the STR regions based on the downloaded mouse sequences from NCBI BLAST program (Rozen and Skaletsky 1999). Parameters were defined in Primer3 to target primers with annealing temperatures of 60 °C and for fixed PCR product sizes (Butler 2005a). All other settings were default settings. AutoDimer software was used to assess primer-dimer interactions and hairpin structures of possible primer combinations to be used in the multiplex (Vallone and Butler 2004). Forward primers were labeled with one of the following fluorescent dyes at the 5′ end: 6FAM™ (blue), VIC™ (green), NED™ (yellow), or PET™ (red) (Applied Biosystems, Foster, CA, USA). In some cases, an additional guanine base (G) or a “PIGtail” sequence (GTTTCTT) was added to the 5′ end of the unlabeled reverse primers to promote complete adenylation (Eurofins MWG Operon, Huntsville, AL, USA) (Brownstein et al. 1996) (Table 1).

### DNA and cell lines

Genomic mouse DNA samples obtained from Jackson Laboratories (Bar Harbor, ME, USA) include 37 inbred mice DNA samples, 1 recombinant inbred mouse sample, and 10 wild-derived mice DNA samples. DNA from 15 wild-caught mice (courtesy of Dr. Michael Nachman from the University of Arizona, USA) collected in Tucson, AZ was used for heterozygosity studies. Genomic DNA from mouse (male and female CD1/ICR), hamster (Syrian golden hamster, Chinese hamster), rat (Fischer, Wistar, Sprague–Dawley), gerbil, pig, baboon, rhesus, and cynomolgus monkey were obtained from Zyagen (San Diego, CA, USA). TN1 cells used in stability studies (courtesy of Dr. Anne Plant from NIST) are a stable transfected NIH3T3 cell line expressing green fluorescent protein (GFP) (parent NIH3T3 cells obtained from the American Type Culture Collection (ATCC, Manassas, VA, USA) in 2003). Cell lines, derived from mouse, Chinese hamster, human, and African green monkey, were obtained from ATCC and their respective growth requirements are described in Table 2. All cells were grown in a humidified 5 % CO_2_ balanced-air atmosphere at 37 °C. For DNA extraction purposes, all cell lines were harvested at passage 3. Adherent cell lines were harvested using 0.25 % trypsin/0.53 mM EDTA solution (ATCC). Trypsin activity was quenched by the addition of an equivalent volume of growth medium (0.1 % soybean inhibitor (Invitrogen) used to neutralize trypsin for MCF 10A cells) and one million cells from each cell line were counted using the Multisizer 3 Coulter Counter (Beckman Coulter, Brea, CA, USA). RAW 264.7 cells were harvested using a cell scraper. The Wizard DNA Extraction kit (Promega, Madison, WI, USA) was used to isolate DNA from harvested cells. DNA was quantified using the Synergy Mx plate reader and Take3 plate (BioTek, Winooski, VT, USA) at an absorbance of 260 nm. To study STR marker stability over increasing passage number, duplicate 25 cm^2^ tissue culture flasks of L929 cells were carried independently, and one million cells were harvested at passage numbers 2, 4, 9, 14, 19, 22, 26, 29, 31, 37, 41, and 44. Duplicate 25 cm^2^ flasks were also carried for NIH3T3 cells which were carried independently, and one million cells were harvested from passage numbers 5, 7, 10, 15, 20, 23, 26, 32, 35, 40, 43, and 45.

**Table 2:** ATCC cell lines and growth media

Cell line	ATCC #	Growth medium	Supplements
NIH3T3	CRL-1658	DMEM	10 % FBS
L929	CCL-1	EMEM	10 % FBS
MC3T3-E1, subclone 4	CRL-2593	MEM	10 % FBS
RAW 264.7	TIB-71	DMEM	10 % FBS
*M. dunni*	CRL-2017	McCoy’s 5A	10 % FBS
P3X63Ag8.653	CRL-1580	RPMI-1640	10 % FBS
HK-PEG-1	CCL-189	IMDM	20 % FBS
Vero	CCL-81	MEM- α	10 % FBS
COS-7	CRL-1651	DMEM	10 % FBS
HEPM	CRL-1486	EMEM	10 % FBS
SK-BR-3	HTB-30	McCoy’s 5A	10 % FBS
MCF 10A	CRL-10317	MECGM	0.4 % bovine pituitary extract, 0.5 μg/mL hydrocortisone, 3 ng/mL human epidermal growth factor, 5 μg/mL insulin
HeLa	CCL-2	EMEM	10 % FBS
CHO-K1	CCL-61	F-12 K	10 % FBS

Dulbecco’s Modified Eagle’s Medium (DMEM) (ATCC), Eagle’s Minimum Essential Medium (EMEM) (ATCC), Minimum Essential Medium (MEM) (Gibco, Carlsbad, CA, USA), Iscove’s Modified Dulbecco’s Medium (IMDM) (ATCC), MEM- α (Invitrogen, Carslbad, CA, USA), RPMI-1640 (ATCC), McCoy’s 5A (ATCC), F-12 K Medium (ATCC), Mammary Epithelial Cell Growth Medium (MECGM) and associated supplements (Lonza, Rockland, ME, USA), fetal bovine serum (FBS) (Invitrogen)

### PCR amplification

PCR amplification was performed on a Veriti thermal cycler (Applied Biosystems). The reaction mixture of 20 μL final volume contained 1 ng of mouse DNA (or 5 ng to10 ng of non-mouse DNA for specificity studies), 1X GeneAmp PCR Gold buffer (Applied Biosystems), 2 mM MgCl_2_ (Applied Biosystems), 250 μM dNTPs (USB Corporation, Cleveland, OH, USA), forward labeled and reverse primers (Table 1), 1U AmpliTaq Gold DNA Polymerase (Applied Biosystems), and 0.16 mg/mL non-acetylated BSA (Invitrogen). PCR conditions for the multiplex assay are as follows: denaturation for 11 min at 95 °C, amplification for 30 cycles of 45 s at 94 °C, 2 min at 59 °C, and 1 min at 72 °C, followed by an extension for 60 min at 60 °C, and a final soak at 25 °C.

### PCR product analysis

Initial unlabeled primers and their respective PCR products were screened by using gel electrophoresis. PCR products (4 μL) were added to the Lonza 5X loading dye (1 μL), loaded onto a 2.2 % agarose Flash Gel (Lonza) and run at 275 V for 5 min. Forward primers generating clean PCR products were ordered with a fluorescent dye at the 5′ end and were tested in monoplex reactions with mouse DNA from Jackson Laboratories, Zyagen, and mouse cell lines. Multiplex reactions were then optimized by varying primer combinations, primer concentrations, DNA concentration, and PCR cycle number. To analyze monoplex and multiplex PCR products, samples were prepared by adding 1 μL of amplified product and 0.3 μL of GeneScan™ 500 LIZ internal size standard (Applied Biosystems) to 8.7 μL of Hi-Di™ (Applied Biosystems) for separation on the 16-capillary ABI 3130*xl* Genetic Analyzer (Applied Biosystems). A five dye matrix was established under the G5 filter with dyes 6FAM, VIC, NED, PET, and LIZ. POP-4™ (Applied Biosystems) was utilized on a 36 cm capillary array (Applied Biosystems) with 1X ACE buffer (Amresco, Solon, OH, USA). Samples were injected electrokinetically for 10 s at 3 kV. The STR alleles were separated at 15 kV at a run temperature of 60 °C. Data from the 3130*xl* was analyzed using the GeneMapper *ID*-*X* v1.1 Software (Applied Biosystems). Bins and panels were created in GeneMapper *ID*-*X* based on fragment length data generated from the fifty-seven mouse profiles using fixed bin allele sizes to determine allele calls. The allele distribution range for the human STR markers (D8S1106 and D4S2408) was previously described (Hill et al. 2008; Almeida et al. 2011) and adjustments were made to the size range to take into account the “PIGtail” sequence that was added to the reverse primers. Calibration of repeat number to allele fragment length was determined by DNA sequencing.

### DNA sequencing

Multiplex primers were used for sequencing STR markers except for three loci (18-3, 9-2, and 12-1) where sequencing primers were used. Table 3 lists the forward and reverse primers used to sequence each marker with corresponding annealing temperatures and amplicon sizes. At least four homozygous samples were sequenced for each STR locus to determine the corresponding number of repeats for each allele. The targeted repeat regions were amplified using 0.15 μM unlabeled forward and reverse primers using the PCR reaction specified in the PCR Amplification section with the following thermal cycling program: denaturation for 10 min at 95 °C, amplification for 35 cycles of 1 min at 94 °C, 1 min at 52–60 °C (annealing temperature specific to individual primers), and 1 min at 72 °C, followed by an extension for 45 min at 60 °C, and a final soak at 25 °C. Samples were treated with 2 μL of ExoSap-IT™ (USB Corporation) per 5 μL of PCR product to remove unincorporated primers and deoxyribonucleotide triphosphates (dNTPs) by incubating samples for 90 min at 37 °C followed by 20 min at 80 °C to inactivate the enzymes. Samples were then sent to Eurofins MWG Operon for sequencing using BigDye^®^ Terminator v3.1 (Applied Biosystems). Resulting profiles were received after data analysis was performed by Eurofins MWG Operon.

**Table 3:** Sequencing primers

STR marker	Primers (5′–3′)	Amplicon size (bp)	T_a_ (°C)
18-3	18-3 F:TCTTTCTCCTTTTGTGTCATGC 18-3 R: GTCAAAGTTGGGGTTACAGAATG*	281–313	54
4-2	4-2 F:AAGCTTCTCTGGCCATTTGA 4-2 R:GTTCATAAACTTCAAGCAATGACA	217–248	57
6-7	6-7 F:AGTCCACCCAGTGCATTCTC 6-7 R:GCATGTGGCTGGTATGCTGTT	333–515	60
9-2	9-2 F:GGCTCTCTCACACCTCATCC* 9-2 R:GTCCATGAATCCAGACATTCC	318–360	60
15-3	15-3F: TCTGGGCGTGTCTGTCATAA 15-3 R:GTTCTCAGGGAGGAGTGTGCT	157–222	60
6-4	6-4 F:TTTGCAACAGCTCAGTTTCC 6-4 R:GAATCGCTGGCAGATCTTAGG	276–311	52
12-1	12-1 F:CAAAATTGTCATTGAACACATGTAA* 12-1 R:GCAATGGTCAAGAAATACTGAAGTACAA	222–259	55
5-5	5-5 F:CGTTTTACCTGGCTGACACA 5-5 R:GATGCTTGCCTGTTCCTAGC	258–298	60
X-1	X-1 F:GGATGGATGGATGGATGAAA X-1 R:GAAGGTATATATCAAGATGGCATTATCA	357–442	54

Sequencing primers listed with their respective amplicon size range (bp) and annealing temperatures (T_a_). Primers that are not included in the multiplex assay (*) were designed for samples that were difficult to sequence with the original primers

### Mixture analysis

Mixture samples containing genomic DNA extracted from NIH3T3, RAW264.7, and HeLa cells were analyzed to assess the capability of the multiplex assay to detect low levels of contamination in NIH3T3 cells. DNA from NIH3T3 and RAW264.7 cells were added to individual reactions with a final concentration of 1 ng of total DNA in the following ratios 1:1, 2:1, 3:1, 5:1, 7:1, 9:1, and 10:1. Reciprocal reactions were also prepared using DNA from RAW264.7 and NIH3T3 cells. The same procedure was repeated using DNA from NIH3T3 and HeLa cells, followed by reciprocal reactions with DNA from HeLa and NIH3T3 cells. PCR amplification and PCR product analysis are described above.

### DNA analysis

The heterozygosity (H) values were calculated by dividing the number of heterozygotes at a locus into the total number of individuals (Weir and Cockerham 1984). The probability of identity (P_I_) was calculated by the summation of the square of the genotype frequencies (Butler 2005b). The probability of a random match (PM) for a full profile was calculated by multiplying the inverse of each genotype frequency for each marker. The coefficient of inbreeding (F), specifically the fixation in a subpopulation compared to the total population (F_ST_) was determined by subtracting the average heterozygosity of the two subpopulations (wild-caught mice and inbred mice samples) from the total heterozygosity, divided by the total heterozygosity (Hartl and Clark 1997; Weir and Cockerham 1984).

## Results and discussion

The mouse primers targeting tetranucleotide repeat markers in the multiplex PCR assay were designed based on the annotated mouse genome from NCBI build 38.1 of *Mus musculus* origin. Fifty-seven genomic mouse DNA samples were tested using the multiplex assay and the designated allele range was determined for each marker, and fragment lengths were correlated to actual number of repeats using sequence analysis (Table 4). The mouse samples were selected to represent the genetic diversity of the mouse family tree (Witmer et al. 2003). To determine the specificity of the multiplex assay we tested DNA from several different species and subspecies of mice, near neighbors, and non-mouse samples.

**Table 4:** Defining STR fragment length with correlating repeat number

STR	Repeat motif	Allele distribution and correlating fragment lengths															
18-3	[ATCT]_n_	137 *12.2*	146 *15*	150 *16*	154 ***17***	158 ***18***	162 ***19***	166 *20*	171 *21*	175 ***22***	179 *23*						
4-2	[GATA]_n_[GATG]_n_[ATAG]_n_	209 *14*	213 *15*	216 *15.3*	217 ***16***	220 *16.3*	221 ***17***	222 *17.1*	224 ***17.3***	225 *18*	228 *18.3*	232 *19.3*	236 ***20.3***	240 ***21.3***	244 ***22.3***	248 *23.3*	
6-7	[CTAT]_n_	333 ***12***	337 *13*	341 ***14***	345 ***15***	349 *16*	352 ***17***	355 ***17.3***	356 ***18***	360 *19*	363 *19.3*	364 *20*	366 *20.1*	368 *21*	376 *23*	377 *23.1*	388 *26*
9-2	[TAGA]_n_[AGAT]_n_	188 *7.1*	192 *8.1*	204 *11.1*	219 ***15***	220 *15.1*	223 ***16***	227 ***17***	231 ***18***	235 *19*							
15-3	[TAGA]_n_	157 *12*	165 *14*	169 ***15***	177 ***17***	181 ***18***	184 ***18.3***	189 ***19.3***	193 *20.3*	196 ***21.3***	200 *22.3*	204 *23.3*	208 ***24.3***	212 *25.3*	216 *26.3*	220 *27.3*	222 *28.1*
6-4	[ATAG]_n_[ATGA][TAGA]_n_	276 *11.2*	285 *13.3*	286 *14*	289 ***14.3***	290 *15*	293 *15.3*	294 ***16***	297 ***16.3***	298 ***17***	301 *17.3*	302 ***18***	307 *19*	311 *20*			
12-1	[AGAT]_n_[GATA]_n_	222 *15*	226 ***16***	227 *16.1*	230 ***17***	231 *17.1*	234 ***18***	235 *18.1*	238 *19*	239 *19.1*	242 *20*	246 *21*	247 ***21.1***	250 *22*	254 *23*	259 *24.1*	
5-5	[TATC]_n_	326 *11*	330 ***12***	334 ***13***	338 ***14***	342 ***15***	346 ***16***	350 ***17***	354 *18*	365 *21*							
X-1	[ATAG]_n_[ATGA]_n_[TAGA]_n_	380 *20*	385 *21*	389 *22*	393 ***23***	397 *24*	401 ***25***	404 *25.3*	405 ***26***	409 ***27***	413 *28*	421 *30*	428 *32*	448 *37*			

Fragment lengths in base pairs represent apparent size based on LIZ GeneScan™ 500 size standard and corresponding number of repeats (italic) was determined by the analysis of 57 mouse DNA samples. The correlation of the allele size and number of repeats was determined based on sequencing data (values highlighted in bold have been sequenced)

### Specificity and sensitivity of the assay

A panel of 57 mouse genomic DNA samples representing species from *M. musculus musculus*, *M. musculus domesticus*, *M. musculus molossinus*, *M. musculus castaneus*, *M. spretus* (Spain), and *M. dunni* were tested with the multiplex PCR primers to determine assay robustness. Full unique profiles amplified in the designated allele range were obtained from the panel for all but the following samples: CAST/EiJ (*M. musculus castaneus*), JF1/Ms (*M. musculus molossinus*), SPRET (*M. spretus*), and *M. dunni* cell line. DNA from CAST and JF1 mice resulted in amplicons for each marker; however, the PCR product was outside of the designated allele range for the 18-3 and 6-7 loci, respectively. Sequencing the CAST mouse DNA revealed that this sample has conserved sequence flanking the repeat region; however, there are fifty-two ATCT repeats at this locus, twenty-nine more than observed in the designated allele range. Due to the additional repeats present in the CAST mouse sample, the amplified product appears between STR markers 4-2 and 6-7. All *M. musculus molossinus* samples resulted in full profiles except for DNA from the JF1 mouse which amplified outside the designated allele range for marker 6-7. The additional thirty-two repeats that JF1 contains at the 6-7 locus may be explained in the origin of *Mus musculus molossinus,* a natural hybrid of *M. m. musculus* and *M. m. castaneus* (Bonhomme and Guenet 1996), the latter shown to deviate from the designated allele range at marker 18-3. DNA from the SPRET mouse (*M. spretus*) results in amplicons that fall outside the designated allele range for the following loci: 18-3, 4-2, 15-3, and X-1. The SPRET sample was sequenced at the 18-3 locus resulting in sixty-six repeats, eleven of which were GTCT repeats embedded within the defined ATCT repeat for this marker. DNA extracted from the *M. dunni* cell line does not amplify at the 6-4 STR marker and falls outside the designated allele range for X-1. Further analysis of DNA from *M.dunni* and SPRET was not continued as their profiles were incomplete using the multiplex assay. Interestingly, CAST and SPRET are mapped together in group 2 in a published mouse family tree (Witmer et al. 2003); however, full profiles within the allele range are observed for the other members in that group including PERC (*M. m. domesticus*), MOLG (*M. m. molossinus*), and MOLF (*M. m. molossinus*).

A panel of rodent and porcine DNA (rat, hamster, gerbil, pig), human cell lines (HeLa, HEPM, SK-BR-3, MCF10A) and nonhuman primate DNA samples (Vero, COS-7, rhesus, baboon, cynomolgus monkey) were tested with the multiplex assay to determine assay specificity. None of these samples resulted in a complete profile using the primers targeting mouse STR markers. DNA from Wistar, Fischer, and Sprague–Dawley rats resulted in a single amplified product in the red dye channel; however, each sample resulted in an amplicon with a fragment length of 219 base pairs. Characteristic stutter peaks associated with polymerase slippage of repeat regions (Walsh et al. 1996) were absent in the rat samples. Lack of stutter peaks and identical amplicon sizes for each rat strain suggests the peak present is most likely a PCR artifact rather than amplification of a repeat region. Amplification products were absent for each mouse STR marker when DNA from human and African green monkey cell lines were tested; however, both cell lines amplified at the human STR markers (D8S1106 and D4S2408) present in the multiplex as expected. No significant amplicons were visible for pig, hamster, or gerbil DNA.

SNP assays, commonly used to type mouse strains, are efficient in discriminating between different strains of mice, but may not be ideal in differentiating between cell lines derived from the same substrain. SNPs are mostly bi-allelic markers whereas STR markers typically have greater than five alleles (Butler 2005b). Using the mouse multiplex assay, unique profiles were obtained for the mouse cell lines listed in Table 5 with the capability of distinguishing between three Balb/c-derived cell lines. There are many conserved alleles between the three Balb/c-derived samples; however, there are sufficient differences resulting in unique profiles for each individual cell line. Two of the Balb/c-derived cell lines, mouse myeloma cells (P3X63Ag8.653) and hybridoma cells (HK-PEG-1), are very similar in their genotype, only varying by one allele at the 9-2 locus. The HK-PEG-1 cell line was produced by fusing P3X63Ag8.653 (myeloma cells originating from a BALB/c mouse) with spleen cells from a BALB/c mouse, explaining why they share so many alleles (Kohler and Milstein 1975). The myeloma cell line is heterozygous at the 9-2 locus whereas the hybridoma cell line is homozygous. To verify the presence of a null allele at the 9-2 marker, a panel of primers was tested with DNA from the hybridoma cells resulting in amplicons ranging from 132 to 244 bp. Homozygote peaks were present in each sample supporting the findings that these two cell lines differ by one allele at this marker.

**Table 5:** Complete genetic profiles of six mouse cell lines

Cell line	Origin	18-3	4-2	6-7	9-2	15-3	6-4	12-1	5-5	X-1
NIH3T3	NIH Swiss	17, 19	19.3, 19.3	12, 12	15, 16	20.3, 20.3	14.3, 14.3	20, 20	14, 15	25, 25
L-929	C3H/An	16, 16	20.3, 20.3	12, 12	15, 15	24.3, 25.3, 26.3	16, 16	16, 16	14, 14	26, 27
MC3T3-E1	C57BL/6	15, 15	20.3, 20.3	17, 17	17, 18	22.3, 22.3	17, 17	17, 17	17, 17	28, 28
RAW264.7	Balb/c	18, 18	22.3, 22.3	12, 12	15, 15	22.3, 22.3	17, 17	16, 16	14, 14	24, 24
P3X63Ag8.653	Balb/c	18, 19	21.3, 21.3	12, 12	15, 16	22.3, 23.3	17, 18	16, 16	13, 14	25, 25
HK-PEG-1	Balb/c	18, 19	21.3, 21.3	12, 12	15, 15	22.3, 23.3	17, 18	16, 16	13, 14	25, 25

The repeat numbers are listed for each locus

Microvariants (an incomplete repeat) are indicated by a decimal point

To test assay sensitivity and determine the lower limits of detection, DNA from NIH3T3, HeLa, and Vero cell lines was diluted from 6 ng to 7.8 pg. A full profile for NIH3T3 cells was obtained using 62 pg of DNA but resulted in a loss of an allele at one mouse STR markers at 31 pg of DNA. The two human STR markers were also tested and resulted in peaks above the analytical threshold (50 relative fluorescent units) for HeLa and Vero cell lines using 62 and 187 pg of DNA but resulted in allelic drop-out at 31 and 93 pg of DNA, respectively. In previous studies, higher concentrations of Vero cell DNA (6 ng) were needed to obtain an STR profile using human STR markers when compared to human DNA (0.5–1 ng) (Almeida et al. 2011). This is consistent with the higher concentrations of Vero DNA needed in this study to amplify efficiently using the human STR markers in the multiplex assay.

### Evaluation of STR markers

Fifteen DNA samples from wild-caught mice were analyzed using the multiplex assay and resulted in unique STR profiles. Heterozygosity values were calculated for these samples and they range from 0.78 to 0.89 (refer to Table 6). To determine the heterozygosity values for the cell lines and inbred mouse samples, a calculation is needed to determine the degree of inbreeding. We compared the fixation index (F_ST_) at each marker to determine the degree of inbreeding between the wild-caught and inbred mouse samples. The calculated F_ST_ values in Table 6 range from 0.005 to 0.06 which is indicative of a very low amount of differentiation between these two subgroups. The probability of identity (P_I_) was also calculated for each marker. For example, the 18-3 locus has a probability of 1 in 5.7 that any two mouse samples would match at this marker. Seven STR loci are located on separate chromosomes except for markers 6-4 and 6-7 which are 90 megabases apart, located on opposite ends of chromosome six, and are considered unlinked. Treating the nine STR markers as though they are located on separate chromosomes, the inverse of the P_I_ for each marker were multiplied to determine the probability of a random match (PM). The probability of a random match using nine STR markers between any two mouse samples is 1 in 5.7 million.

**Table 6:** Heterozygosity, probability of identity, and fixation index values calculated for each mouse STR marker

STR marker	H(w)	H(i)	H(t)	P_I_	F_ST_
18-3	0.846	0.800	0.842	0.175	0.023
4-2	0.792	0.808	0.849	0.150	0.057
6-7	0.863	0.744	0.818	0.181	0. 017
9-2	0.783	0.531	0.700	0.300	0.060
15-3	0.891	0.847	0.882	0.117	0.014
6-4	0.818	0.779	0.837	0.162	0.045
12-1	0.815	0.773	0.823	0.176	0.034
5-5	0.780	0.787	0.803	0.197	0.023
X-1	0.830	0.795	0.817	0.182	0.005

*H(w)* heterozygosity of wild-caught mice, *H(i)* heterozygosity of inbred mice/cell lines, *H(t)* heterozygosity of total, *P*_*I*_ probability of identity, *F*_*ST*_ fixation index

### Mixture analysis

**Fig. 1:**
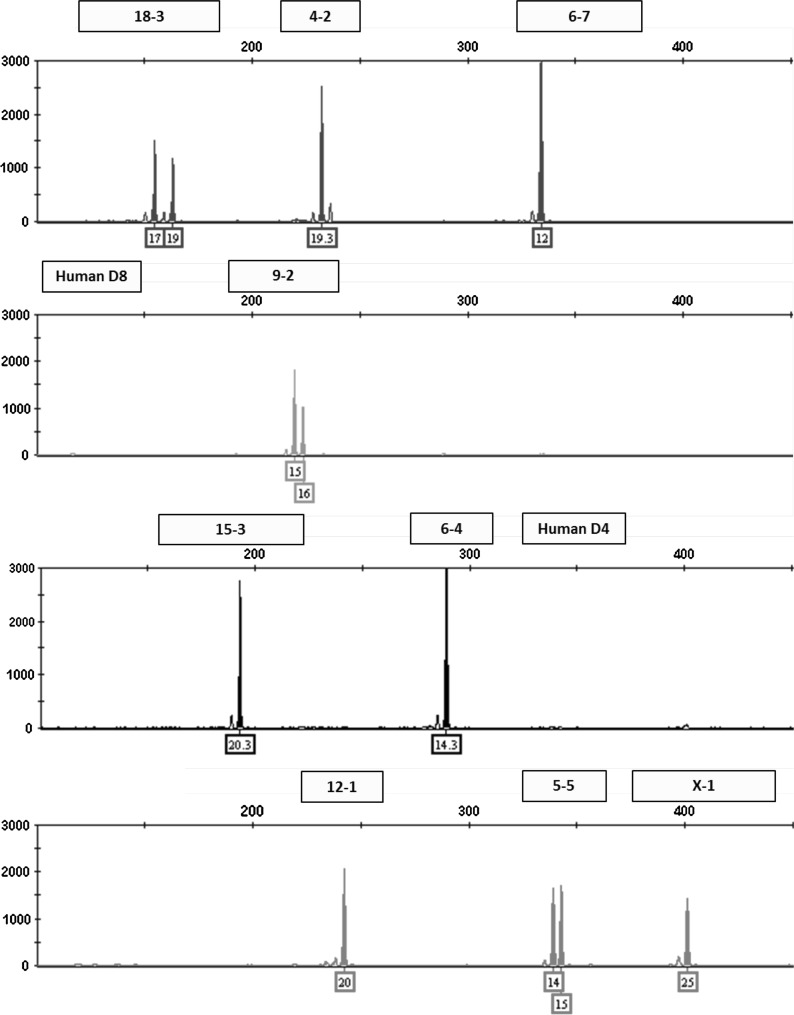
Genetic profile of the NIH3T3 cell line using the mouse multiplex assay (1 ng DNA)

**Fig. 2:**
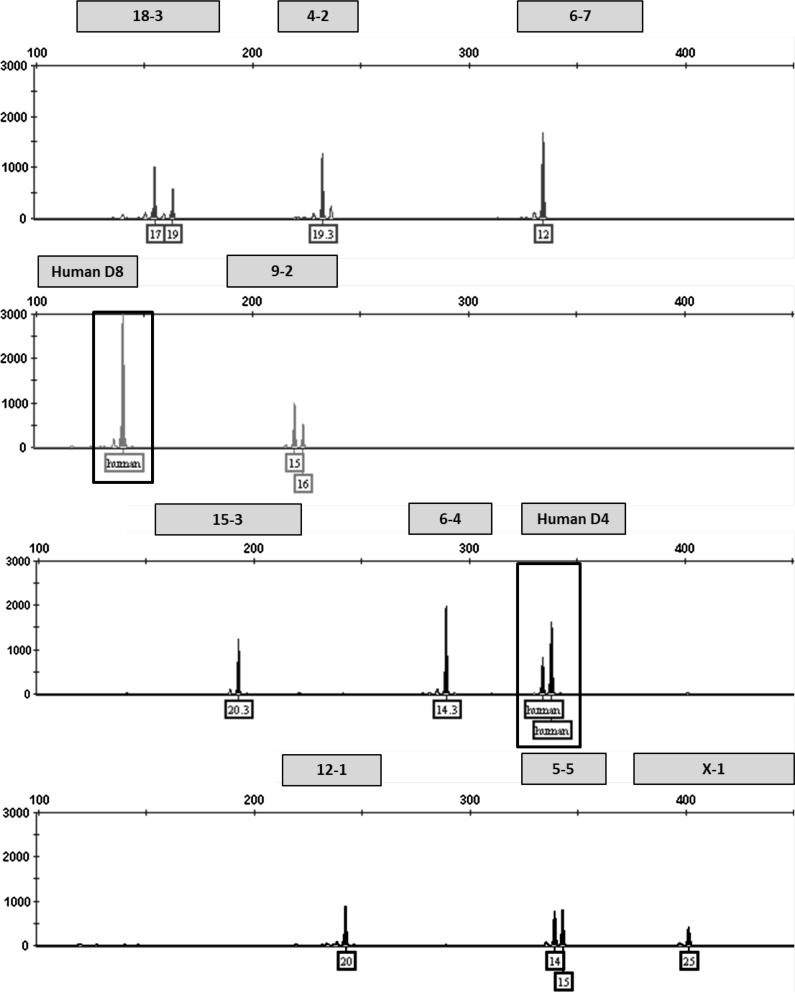
Human contaminant detected in NIH3T3 STR profile (1:1 ratio of DNA from NIH3T3 and HeLa cell lines)

**Fig. 3:**
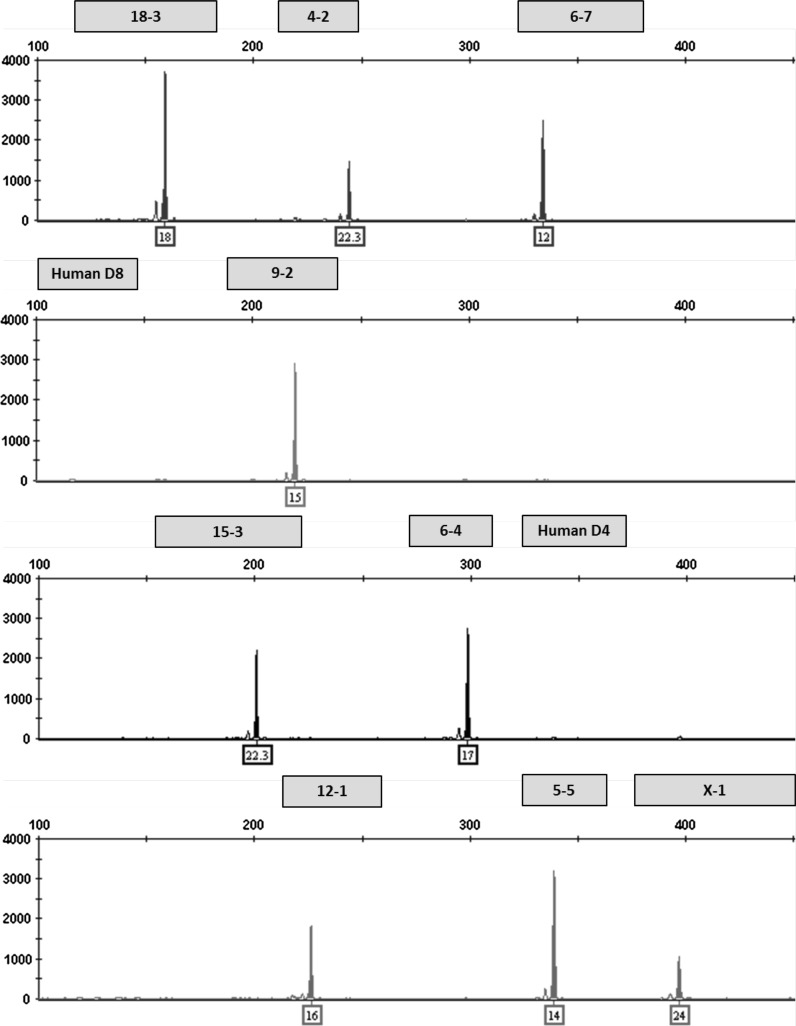
Genetic profile of the RAW 264.7 cell line using the mouse multiplex assay (1 ng DNA)

**Fig. 4:**
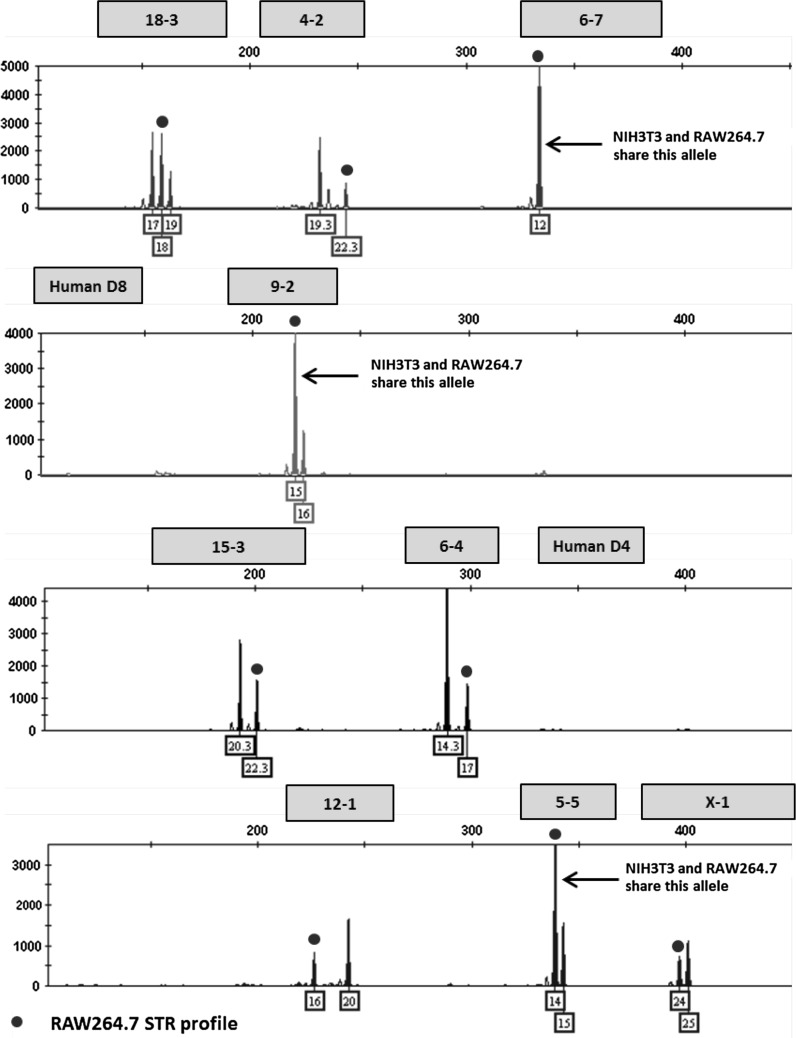
A mixture of NIH3T3 and RAW264.7 mouse cell lines detected using the mouse multiplex assay

This multiplex assay was designed to detect human or African green monkey cell line contamination of mouse cells by incorporating two human STR markers that amplify outside the designated allele ranges for the nine mouse STR markers. Mixture ratios ranging from 1:1 to 10:1 of NIH3T3 and HeLa DNA were tested to model contamination scenarios. An electropherogram depicting a pure NIH3T3 STR profile is shown in Fig. 1. A 1:1 ratio of NIH3T3 and HeLa DNA is shown in Fig. 2. Even at the lowest dilution of HeLa DNA (90 pg), human STR markers were detected above the analytical threshold. The assay can also be used to detect a mixture of multiple mouse cell lines. An electropherogram depicting a pure RAW264.7 STR profile is shown in Fig. 3. Mixture ratios ranging from 1:1 to 10:1 of NIH3T3 and RAW264.7 DNA were tested and full profiles of both cell lines were present even at the lowest DNA dilution (90 pg). Figure 4 shows a 1:1 mixture of the two mouse cell lines.

The majority of mouse cell lines are derived from inbred mice resulting in alleles that are mostly homozygous in nature (Green 1968; Russell 1996). For example, the RAW 264.7 mouse cell line is homozygous at each STR marker (Table 5). Multiple alleles present at each locus could indicate a mixed population of cells. Triallelic patterns have been observed in some human cell lines at a particular locus, which may or may not be equal in intensity (Butler 2005b). The L929 cell line appears to have three alleles with similar peak height intensities at the 15-3 marker and each allele is four base pairs or one repeat apart. Since most of the mouse samples tested were homozygous for the majority of the markers, a panel of primers targeting the 15-3 locus were tested in monoplex with DNA from L929 cells. The amplicons ranged from 210 to 435 base pairs in length and each resulted in three alleles that were four bases apart with very little peak height imbalance. The evidence supports a true triallelic pattern at the 15-3 marker.

### STR marker stability

While alteration of genetic profiles of some cancer cell lines has been observed previously at high passage numbers (Parson et al. 2005), other studies show STR stability over high passage numbers in some human cancer cell lines (Chiong et al. 2009) and in African green monkey cell lines (Almeida et al. 2011) indicating stability may be cell line dependent. To test the stability of the mouse STR markers in this assay, L929 and NIH3T3 cell lines were carried independently and in duplicate flasks up to passage 44 and 45, respectively. Genotypes were determined and standard deviations were calculated for each locus representing the variations in fragment lengths over all passage numbers. The NIH3T3 cell line resulted in the lowest standard deviation values (0.02–0.05) for each locus. The L929 cell line resulted in standard deviations ranging from 0.05 to 0.14. The STR markers with the highest standard deviations in L929 cells are 6-7 (0.14) and 5-5 (0.13). In both the NIH3T3 and L929 cell lines, even the highest standard deviation values did not result in an allele repeat number change indicating stable STR profiles at high passage numbers. The changes in fragment lengths for each marker over the passage period were not significant enough to change the allele calls and the variability in the amplicon sizes fell within the range of the instrument fluctuation. Identical DNA samples were tested on three different days using the same instrument and the variation in fragment length was ±0.3 base pairs.

In addition to stability of the STR profile for NIH3T3 cells over time, we were also interested in evaluating profile stability after transfection procedures. The TN1 cell line, derived from NIH3T3 cells obtained from ATCC in 2003 and engineered to express the gene for green fluorescent protein, was analyzed using the multiplex assay and resulted in identical STR profiles for both TN1 and recently obtained NIH3T3 cells. These data support the findings that the STR markers are stable over time in transfected NIH3T3 cell lines.

In conclusion, the mouse multiplex assay described in this report can be used to identify cell lines derived from *M. musculus musculus* and *M. musculus domesticus* species. The assay is also useful in identifying *M. musculus molossinus* and *M. musculus castaneus* species which amplify at each locus, but in some instances failed to fall within the designated allele range for one of the STR markers. This assay is not recommended for genotyping mouse cell lines derived from *M. spretus* (amplicons fall outside the designated allele range for four STR markers) or *M. dunni* which fails to amplify at the 6-4 locus. Stability studies show the mouse STR markers are stable with high passage numbers and the STR profiles remain unchanged after transfection procedures in the TN1 cell line. Although the STR markers are stable up through passages 44–45, best practices suggest genotyping samples at low passage numbers (Reid 2011). The power of discrimination based on the probability of a random match is 1 in 5.7 million using the nine STR markers in the multiplex assay. This assay can be used to identify both human and African green monkey cell line contaminants using the two human STR markers incorporated in the multiplex assay in addition to detecting mixtures of mouse cell lines. The targeted tetranucleotide repeat regions in the mouse genome result in unique individual profiles making this assay more sensitive and specific than those that are currently available. The requirement of cell line authentication is becoming more routine, and this assay provides a reliable method to genotype mouse cell lines.
